# *N*-(3-Chloro­phen­yl)-2,4-dimethyl­benzene­sulfonamide

**DOI:** 10.1107/S1600536811029400

**Published:** 2011-07-30

**Authors:** K. Shakuntala, Sabine Foro, B. Thimme Gowda

**Affiliations:** a Department of Chemistry, Mangalore University, Mangalagangotri 574 199, Mangalore, India; b Institute of Materials Science, Darmstadt University of Technology, Petersenstrasse 23, D-64287 Darmstadt, Germany

## Abstract

In the mol­ecule of the title compound, C_14_H_14_ClNO_2_S, the N—H bond is in a *syn* position with respect to the *meta*-Cl atom in the aniline ring. The mol­ecule is twisted about the N—S bond with a C—SO_2_—NH—C torsion angle of 44.55 (17)°. The two aromatic rings are inclined relative to each other by 66.2 (1)°. In the crystal, N—H⋯O hydrogen bonds link the mol­ecules into infinite chains parallel to the *b* axis.

## Related literature

For hydrogen bonding modes of sulfonamides, see: Adsmond & Grant (2001 ▶). For studies on the effects of substituents on the structures and other aspects of *N*-(ar­yl)-amides, see: Arjunan *et al.* (2004 ▶); Gowda *et al.* (1999 ▶), on *N*-(ar­yl)methane­sulfonamides, see: Gowda *et al.* (2007 ▶) and on *N*-(ar­yl)aryl­sulfonamides, see: Gelbrich *et al.* (2007 ▶); Gowda *et al.* (2010 ▶); Perlovich *et al.* (2006 ▶). For the preparation of the title compound, See: Savitha & Gowda (2006 ▶)
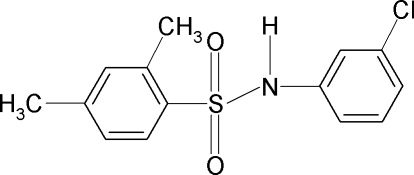


## Experimental

### 

#### Crystal data

C_14_H_14_ClNO_2_S
*M* *_r_* = 295.77
Monoclinic, 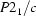

*a* = 9.0385 (7) Å
*b* = 9.7830 (7) Å
*c* = 15.897 (1) Å
β = 97.284 (7)°
*V* = 1394.33 (17) Å^3^
*Z* = 4
Mo *K*α radiation
μ = 0.42 mm^−1^
*T* = 293 K
0.44 × 0.40 × 0.32 mm

#### Data collection

Oxford Diffraction Xcalibur diffractometer with a Sapphire CCD detector
Absorption correction: multi-scan (*CrysAlis RED*; Oxford Diffraction, 2009 ▶) *T*_min_ = 0.837, *T*_max_ = 0.877
5245 measured reflections
2841 independent reflections
2438 reflections with *I* > 2σ(*I*)
*R*_int_ = 0.009

#### Refinement

*R*[*F* ^2^ > 2σ(*F* ^2^)] = 0.036
*wR*(*F* ^2^) = 0.103
*S* = 1.06
2841 reflections
177 parameters
1 restraint
H atoms treated by a mixture of independent and constrained refinement
Δρ_max_ = 0.28 e Å^−3^
Δρ_min_ = −0.36 e Å^−3^

### 

Data collection: *CrysAlis CCD* (Oxford Diffraction, 2009 ▶); cell refinement: *CrysAlis RED* (Oxford Diffraction, 2009 ▶); data reduction: *CrysAlis RED*; program(s) used to solve structure: *SHELXS97* (Sheldrick, 2008 ▶); program(s) used to refine structure: *SHELXL97* (Sheldrick, 2008 ▶); molecular graphics: *PLATON* (Spek, 2009 ▶); software used to prepare material for publication: *SHELXL97*.

## Supplementary Material

Crystal structure: contains datablock(s) I, global. DOI: 10.1107/S1600536811029400/bt5582sup1.cif

Structure factors: contains datablock(s) I. DOI: 10.1107/S1600536811029400/bt5582Isup2.hkl

Supplementary material file. DOI: 10.1107/S1600536811029400/bt5582Isup3.cml

Additional supplementary materials:  crystallographic information; 3D view; checkCIF report

## Figures and Tables

**Table 1:** Hydrogen-bond geometry (Å, °)

*D*—H⋯*A*	*D*—H	H⋯*A*	*D*⋯*A*	*D*—H⋯*A*
N1—H1*N*⋯O1^i^	0.84 (2)	2.32 (2)	3.117 (2)	158 (2)

Symmetry code: (i) 
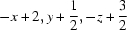
.

**Fig. 1.**
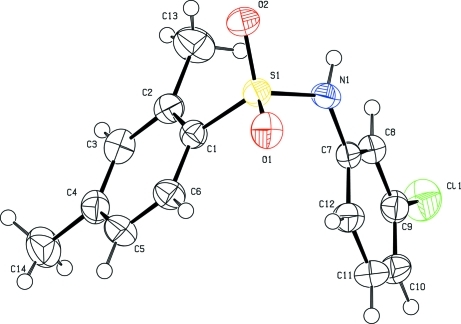
Molecular structure of (I), showing the atom labelling scheme and displacement ellipsoids are drawn at the 50% probability level.

**Fig. 2.**
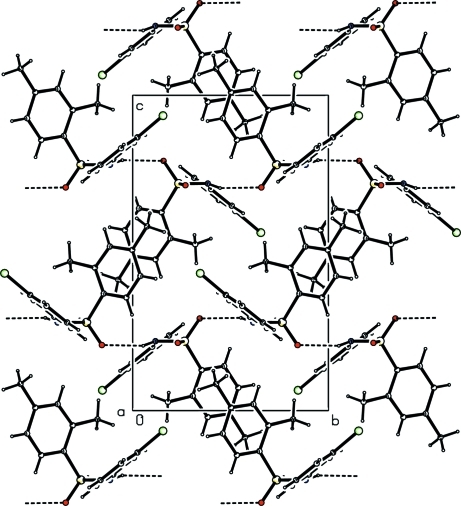
Molecular packing of (I) with hydrogen bonding shown as dashed lines.

## supplementary crystallographic information

### Comment

The amide and sulfonamide moieties are the constituents of many biologically important compounds. The hydrogen bonding preferences of sulfonamides have been investigated (Adsmond & Grant, 2001). As part of our work on the substituent effects on the structures and other aspects of *N*-(aryl)-amides (Arjunan *et al.*, 2004; Gowda *et al.*, 1999), *N*-(aryl)-methanesulfonamides (Gowda *et al.*, 2007) and *N*-(aryl)-arylsulfonamides (Gowda *et al.*, 2010), in the present work, the crystal structure of *N*-(3-chlorophenyl)-2,4-dimethylbenzene- sulfonamide (I) has been determined (Fig. 1). The conformation of the N—C bond in the C—SO_2_—NH—C segment has *gauche* torsions with respect to the S═O bonds. The molecule is bent at the S atom with the C1—SO_2_—NH—C7 torsion angle of 44.6 (2)°, compared to the values of -54.9 (2)° in *N*-(2-chlorophenyl)-2,4-dimethylbenzenesulfonamide (II) (Gowda *et al.*, 2010). Further, the conformation of the N—H bond in (I) is *syn* to the *meta*-chloro group in the anilino ring.

The two benzene rings in (I) are tilted relative to each other by 75.7 (1)°, compared to the value of 66.2 (1)° (II). The other bond parameters in (I) are similar to those observed in (II) and other aryl sulfonamides (Perlovich *et al.*, 2006; Gelbrich *et al.*, 2007).

The crystal packing of molecules in (I) *via* N—H···O(S) hydrogen bonds (Table 1) is shown in Fig.2.

### Experimental

The solution of 1,3-xylene (1,3-dimethylbenzene) (10 ml) in chloroform (40 ml) was treated dropwise with chlorosulfonic acid (25 ml) at 0 ° C. After the initial evolution of hydrogen chloride subsided, the reaction mixture was brought to room temperature and poured into crushed ice in a beaker. The chloroform layer was separated, washed with cold water and allowed to evaporate slowly. The residual 2,4-dimethylbenzenesulfonylchloride was treated with 3-chloroaniline in the stoichiometric ratio and boiled for ten minutes. The reaction mixture was then cooled to room temperature and added to ice cold water (100 cc). The resultant solid *N*-(3-chlorophenyl)-2,4-dimethylbenzenesulfonamide was filtered under suction and washed thoroughly with cold water. It was then recrystallized to constant melting point from dilute ethanol. The purity of the compound was checked and characterized by recording its infrared and NMR spectra (Savitha & Gowda, 2006).

The prism like colourless single crystals used in X-ray diffraction studies were grown in ethanolic solution by a slow evaporation at room temperature.

### Refinement

The H atom of the NH group was located in a difference map and later restrained to the distance N—H = 0.86 (2) Å. The other H atoms were positioned with idealized geometry using a riding model with the aromatic C—H = 0.93Å and methyl C—H = 0.96 Å, All H atoms were refined with isotropic displacement parameters  set to 1.2 times of the *U*_eq_ of the parent atom.

### Crystal data

**Table:**

C_14_H_14_ClNO_2_S	*F*(000) = 616
*M**_r_* = 295.77	*D*_x_ = 1.409 Mg m^−^^3^
Monoclinic, *P*2_1_/*c*	Mo *K*α radiation, λ = 0.71073 Å
Hall symbol: -P 2ybc	Cell parameters from 2954 reflections
*a* = 9.0385 (7) Å	θ = 2.6–27.8°
*b* = 9.7830 (7) Å	µ = 0.42 mm^−^^1^
*c* = 15.897 (1) Å	*T* = 293 K
β = 97.284 (7)°	Prism, colourless
*V* = 1394.33 (17) Å^3^	0.44 × 0.40 × 0.32 mm
*Z* = 4	

### Data collection

**Table:**

Oxford Diffraction Xcalibur diffractometer with a Sapphire CCD detector	2841 independent reflections
Radiation source: fine-focus sealed tube	2438 reflections with *I* > 2σ(*I*)
graphite	*R*_int_ = 0.009
Rotation method data acquisition using ω and φ scans	θ_max_ = 26.4°, θ_min_ = 2.6°
Absorption correction: multi-scan (*CrysAlis RED*; Oxford Diffraction, 2009)	*h* = −11→5
*T*_min_ = 0.837, *T*_max_ = 0.877	*k* = −12→5
5245 measured reflections	*l* = −19→19

### Refinement

**Table:**

Refinement on *F*^2^	Primary atom site location: structure-invariant direct methods
Least-squares matrix: full	Secondary atom site location: difference Fourier map
*R*[*F*^2^ > 2σ(*F*^2^)] = 0.036	Hydrogen site location: inferred from neighbouring sites
*wR*(*F*^2^) = 0.103	H atoms treated by a mixture of independent and constrained refinement
*S* = 1.06	*w* = 1/[σ^2^(*F*_o_^2^) + (0.0568*P*)^2^ + 0.5258*P*] where *P* = (*F*_o_^2^ + 2*F*_c_^2^)/3
2841 reflections	(Δ/σ)_max_ = 0.001
177 parameters	Δρ_max_ = 0.28 e Å^−^^3^
1 restraint	Δρ_min_ = −0.36 e Å^−^^3^

### Special details

**Table:**

Geometry. All e.s.d.'s (except the e.s.d. in the dihedral angle between two l.s. planes) are estimated using the full covariance matrix. The cell e.s.d.'s are taken into account individually in the estimation of e.s.d.'s in distances, angles and torsion angles; correlations between e.s.d.'s in cell parameters are only used when they are defined by crystal symmetry. An approximate (isotropic) treatment of cell e.s.d.'s is used for estimating e.s.d.'s involving l.s. planes.
Refinement. Refinement of *F*^2^ against ALL reflections. The weighted *R*-factor *wR* and goodness of fit *S* are based on *F*^2^, conventional *R*-factors *R* are based on *F*, with *F* set to zero for negative *F*^2^. The threshold expression of *F*^2^ > σ(*F*^2^) is used only for calculating *R*-factors(gt) *etc*. and is not relevant to the choice of reflections for refinement. *R*-factors based on *F*^2^ are statistically about twice as large as those based on *F*, and *R*- factors based on ALL data will be even larger.

### Fractional atomic coordinates and isotropic or equivalent isotropic displacement parameters (Å^2^)

**Table:**

	*x*	*y*	*z*	*U*_iso_*/*U*_eq_	
C1	0.89077 (18)	0.14785 (17)	0.62875 (11)	0.0313 (4)	
C2	0.90726 (19)	0.19319 (18)	0.54672 (11)	0.0359 (4)	
C3	0.8365 (2)	0.1167 (2)	0.47948 (12)	0.0407 (4)	
H3	0.8471	0.1439	0.4245	0.049*	
C4	0.7512 (2)	0.0022 (2)	0.49045 (12)	0.0413 (4)	
C5	0.7383 (2)	−0.0396 (2)	0.57271 (13)	0.0453 (5)	
H5	0.6825	−0.1170	0.5816	0.054*	
C6	0.8074 (2)	0.03244 (19)	0.64125 (12)	0.0385 (4)	
H6	0.7980	0.0035	0.6960	0.046*	
C7	0.73064 (18)	0.40051 (18)	0.70252 (11)	0.0328 (4)	
C8	0.67615 (19)	0.50629 (19)	0.64977 (12)	0.0371 (4)	
H8	0.7410	0.5643	0.6259	0.045*	
C9	0.5229 (2)	0.5243 (2)	0.63306 (12)	0.0402 (4)	
C10	0.4248 (2)	0.4403 (2)	0.66791 (13)	0.0459 (5)	
H10	0.3224	0.4542	0.6563	0.055*	
C11	0.4810 (2)	0.3354 (2)	0.72028 (14)	0.0467 (5)	
H11	0.4157	0.2778	0.7440	0.056*	
C12	0.6335 (2)	0.3141 (2)	0.73829 (13)	0.0410 (4)	
H12	0.6703	0.2430	0.7738	0.049*	
C13	0.9916 (3)	0.3198 (2)	0.52740 (15)	0.0575 (6)	
H13A	1.0910	0.3159	0.5569	0.069*	
H13B	0.9415	0.3991	0.5455	0.069*	
H13C	0.9960	0.3252	0.4675	0.069*	
C14	0.6723 (3)	−0.0736 (3)	0.41520 (15)	0.0614 (6)	
H14A	0.7398	−0.0875	0.3740	0.074*	
H14B	0.5881	−0.0212	0.3904	0.074*	
H14C	0.6387	−0.1606	0.4334	0.074*	
N1	0.88789 (16)	0.38382 (16)	0.72030 (11)	0.0376 (4)	
H1N	0.943 (2)	0.4465 (19)	0.7057 (13)	0.045*	
O1	0.93966 (15)	0.15746 (14)	0.79211 (8)	0.0437 (3)	
O2	1.12517 (14)	0.26929 (15)	0.71487 (9)	0.0469 (4)	
Cl1	0.45335 (6)	0.65483 (6)	0.56450 (4)	0.06029 (19)	
S1	0.97232 (4)	0.23553 (4)	0.72011 (3)	0.03353 (14)	

### Atomic displacement parameters (Å^2^)

**Table:**

	*U*^11^	*U*^22^	*U*^33^	*U*^12^	*U*^13^	*U*^23^
C1	0.0310 (8)	0.0294 (8)	0.0333 (8)	0.0027 (6)	0.0027 (6)	0.0007 (7)
C2	0.0360 (9)	0.0325 (9)	0.0395 (9)	0.0025 (7)	0.0061 (7)	0.0050 (7)
C3	0.0440 (10)	0.0439 (10)	0.0339 (9)	0.0081 (8)	0.0037 (7)	0.0030 (8)
C4	0.0393 (9)	0.0408 (10)	0.0427 (10)	0.0060 (8)	0.0005 (8)	−0.0079 (8)
C5	0.0475 (11)	0.0349 (10)	0.0534 (12)	−0.0084 (8)	0.0064 (9)	−0.0023 (8)
C6	0.0422 (9)	0.0347 (9)	0.0386 (9)	−0.0038 (8)	0.0055 (7)	0.0041 (7)
C7	0.0273 (8)	0.0326 (8)	0.0384 (9)	−0.0005 (7)	0.0042 (7)	−0.0097 (7)
C8	0.0346 (9)	0.0342 (9)	0.0432 (10)	−0.0012 (7)	0.0074 (7)	−0.0047 (8)
C9	0.0373 (9)	0.0402 (10)	0.0419 (10)	0.0053 (8)	0.0006 (7)	−0.0080 (8)
C10	0.0284 (9)	0.0547 (12)	0.0540 (12)	0.0006 (8)	0.0032 (8)	−0.0118 (10)
C11	0.0338 (9)	0.0515 (12)	0.0563 (12)	−0.0088 (8)	0.0110 (9)	−0.0037 (10)
C12	0.0367 (9)	0.0386 (10)	0.0483 (11)	−0.0019 (8)	0.0077 (8)	0.0010 (8)
C13	0.0710 (14)	0.0507 (13)	0.0517 (13)	−0.0151 (11)	0.0110 (11)	0.0133 (10)
C14	0.0632 (14)	0.0641 (15)	0.0537 (13)	−0.0016 (12)	−0.0052 (11)	−0.0189 (12)
N1	0.0270 (7)	0.0298 (8)	0.0558 (10)	−0.0027 (6)	0.0037 (7)	−0.0041 (7)
O1	0.0500 (8)	0.0452 (8)	0.0348 (7)	0.0053 (6)	0.0014 (6)	0.0012 (6)
O2	0.0261 (6)	0.0531 (8)	0.0605 (9)	0.0008 (6)	0.0013 (6)	−0.0101 (7)
Cl1	0.0576 (3)	0.0566 (3)	0.0637 (4)	0.0163 (3)	−0.0036 (3)	0.0070 (3)
S1	0.0284 (2)	0.0337 (2)	0.0376 (2)	0.00265 (16)	0.00062 (16)	−0.00264 (17)

### Geometric parameters (Å, °)

**Table:**

C1—C6	1.386 (2)	C9—C10	1.376 (3)
C1—C2	1.403 (2)	C9—Cl1	1.744 (2)
C1—S1	1.7655 (17)	C10—C11	1.378 (3)
C2—C3	1.392 (3)	C10—H10	0.9300
C2—C13	1.506 (3)	C11—C12	1.388 (3)
C3—C4	1.383 (3)	C11—H11	0.9300
C3—H3	0.9300	C12—H12	0.9300
C4—C5	1.389 (3)	C13—H13A	0.9600
C4—C14	1.508 (3)	C13—H13B	0.9600
C5—C6	1.379 (3)	C13—H13C	0.9600
C5—H5	0.9300	C14—H14A	0.9600
C6—H6	0.9300	C14—H14B	0.9600
C7—C8	1.383 (3)	C14—H14C	0.9600
C7—C12	1.391 (3)	N1—S1	1.6394 (15)
C7—N1	1.423 (2)	N1—H1N	0.844 (15)
C8—C9	1.388 (2)	O1—S1	1.4375 (14)
C8—H8	0.9300	O2—S1	1.4331 (13)
			
C6—C1—C2	120.98 (16)	C11—C10—H10	120.6
C6—C1—S1	117.11 (13)	C10—C11—C12	121.16 (18)
C2—C1—S1	121.91 (13)	C10—C11—H11	119.4
C3—C2—C1	116.81 (16)	C12—C11—H11	119.4
C3—C2—C13	118.72 (17)	C11—C12—C7	119.09 (18)
C1—C2—C13	124.44 (18)	C11—C12—H12	120.5
C4—C3—C2	123.20 (17)	C7—C12—H12	120.5
C4—C3—H3	118.4	C2—C13—H13A	109.5
C2—C3—H3	118.4	C2—C13—H13B	109.5
C3—C4—C5	118.18 (17)	H13A—C13—H13B	109.5
C3—C4—C14	120.90 (19)	C2—C13—H13C	109.5
C5—C4—C14	120.92 (19)	H13A—C13—H13C	109.5
C6—C5—C4	120.60 (18)	H13B—C13—H13C	109.5
C6—C5—H5	119.7	C4—C14—H14A	109.5
C4—C5—H5	119.7	C4—C14—H14B	109.5
C5—C6—C1	120.23 (17)	H14A—C14—H14B	109.5
C5—C6—H6	119.9	C4—C14—H14C	109.5
C1—C6—H6	119.9	H14A—C14—H14C	109.5
C8—C7—C12	120.54 (16)	H14B—C14—H14C	109.5
C8—C7—N1	118.44 (16)	C7—N1—S1	123.69 (12)
C12—C7—N1	121.01 (17)	C7—N1—H1N	118.4 (15)
C7—C8—C9	118.76 (17)	S1—N1—H1N	110.5 (15)
C7—C8—H8	120.6	O2—S1—O1	117.75 (9)
C9—C8—H8	120.6	O2—S1—N1	104.39 (8)
C10—C9—C8	121.73 (18)	O1—S1—N1	109.03 (8)
C10—C9—Cl1	119.26 (15)	O2—S1—C1	111.66 (8)
C8—C9—Cl1	119.00 (15)	O1—S1—C1	106.89 (8)
C9—C10—C11	118.72 (17)	N1—S1—C1	106.59 (8)
C9—C10—H10	120.6		
			
C6—C1—C2—C3	−0.1 (3)	C8—C9—C10—C11	−0.4 (3)
S1—C1—C2—C3	−179.26 (13)	Cl1—C9—C10—C11	178.36 (15)
C6—C1—C2—C13	177.94 (19)	C9—C10—C11—C12	0.3 (3)
S1—C1—C2—C13	−1.2 (3)	C10—C11—C12—C7	−0.1 (3)
C1—C2—C3—C4	1.0 (3)	C8—C7—C12—C11	0.0 (3)
C13—C2—C3—C4	−177.20 (19)	N1—C7—C12—C11	179.00 (17)
C2—C3—C4—C5	−1.3 (3)	C8—C7—N1—S1	−136.09 (15)
C2—C3—C4—C14	177.68 (18)	C12—C7—N1—S1	44.9 (2)
C3—C4—C5—C6	0.8 (3)	C7—N1—S1—O2	162.85 (15)
C14—C4—C5—C6	−178.21 (19)	C7—N1—S1—O1	−70.50 (17)
C4—C5—C6—C1	0.0 (3)	C7—N1—S1—C1	44.55 (17)
C2—C1—C6—C5	−0.3 (3)	C6—C1—S1—O2	133.91 (14)
S1—C1—C6—C5	178.83 (15)	C2—C1—S1—O2	−46.93 (17)
C12—C7—C8—C9	−0.2 (3)	C6—C1—S1—O1	3.82 (16)
N1—C7—C8—C9	−179.16 (16)	C2—C1—S1—O1	−177.03 (14)
C7—C8—C9—C10	0.4 (3)	C6—C1—S1—N1	−112.67 (14)
C7—C8—C9—Cl1	−178.42 (13)	C2—C1—S1—N1	66.48 (16)

### Hydrogen-bond geometry (Å, °)

**Table:**

*D*—H···*A*	*D*—H	H···*A*	*D*···*A*	*D*—H···*A*
N1—H1N···O1^i^	0.84 (2)	2.32 (2)	3.117 (2)	158 (2)

Symmetry codes: (i) −*x*+2, *y*+1/2, −*z*+3/2.
